# Supplementary material for: Rheological engineering of perovskite suspension toward high-resolution X-ray flat-panel detector
Source: Nat Commun. 2023 Oct 27;14:6865. doi: 10.1038/s41467-023-42616-5 (PMC10611698; doi:10.1038/s41467-023-42616-5)
Supplement: Supplementary file 1 — Supplementary Information [file 41467_2023_42616_MOESM1_ESM.pdf]

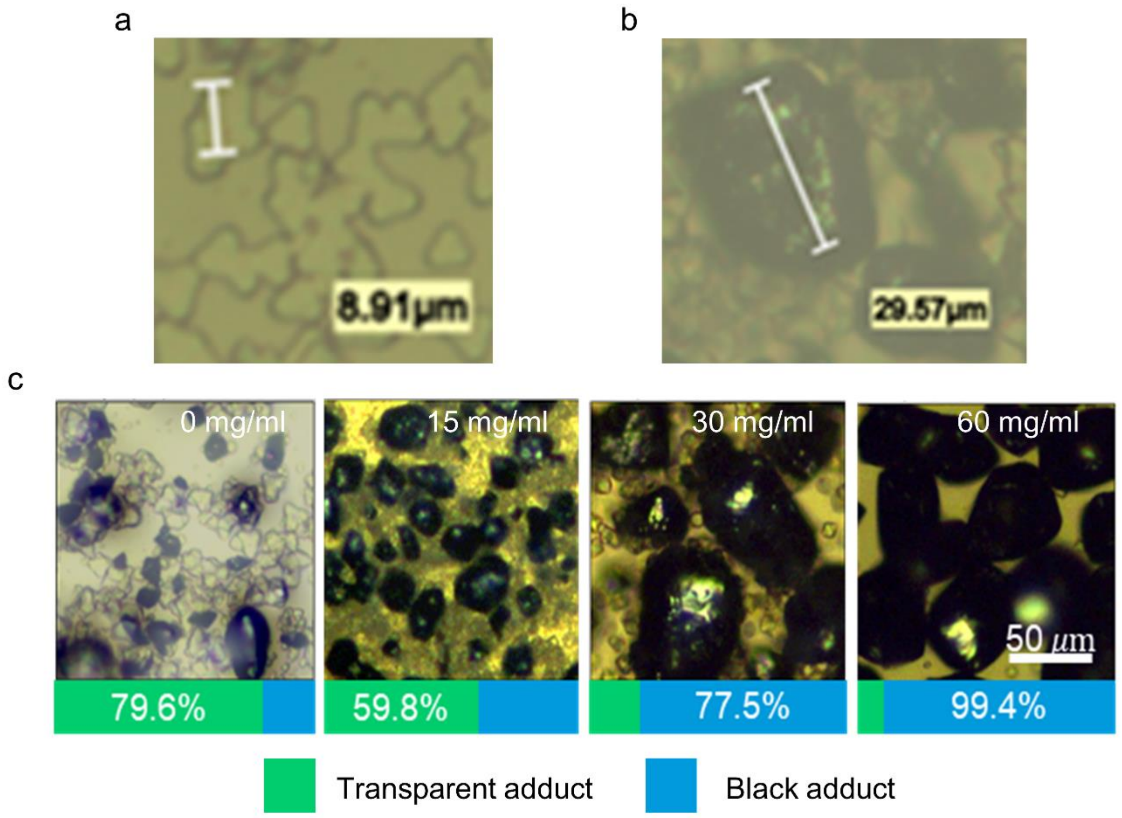

Figure S1. The adduct particles in perovskite suspension. (a) Transparent adduct. (b) Black adduct. (c) Adducts in suspensions with different extra addition of MAI.

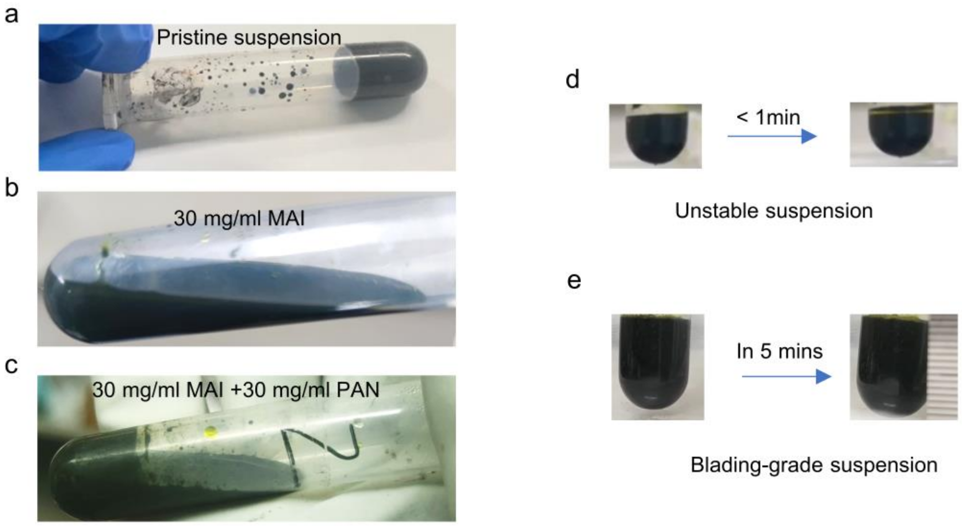

Figure S2. The exhibition of flowability and stability of perovskite suspension. (a) Pristine suspension, (b) Pristine suspension with 30 mg/ml MAI addition, (c) Pristine suspension with 30 mg/ml MAI and 30 mg/ml PAN addition, in horizontal position. (d) Unstable suspension (30 mg/ml MAI addition). (e) Blading-grade suspension (30 mg/ml MAI and 30 mg/ml PAN addition).

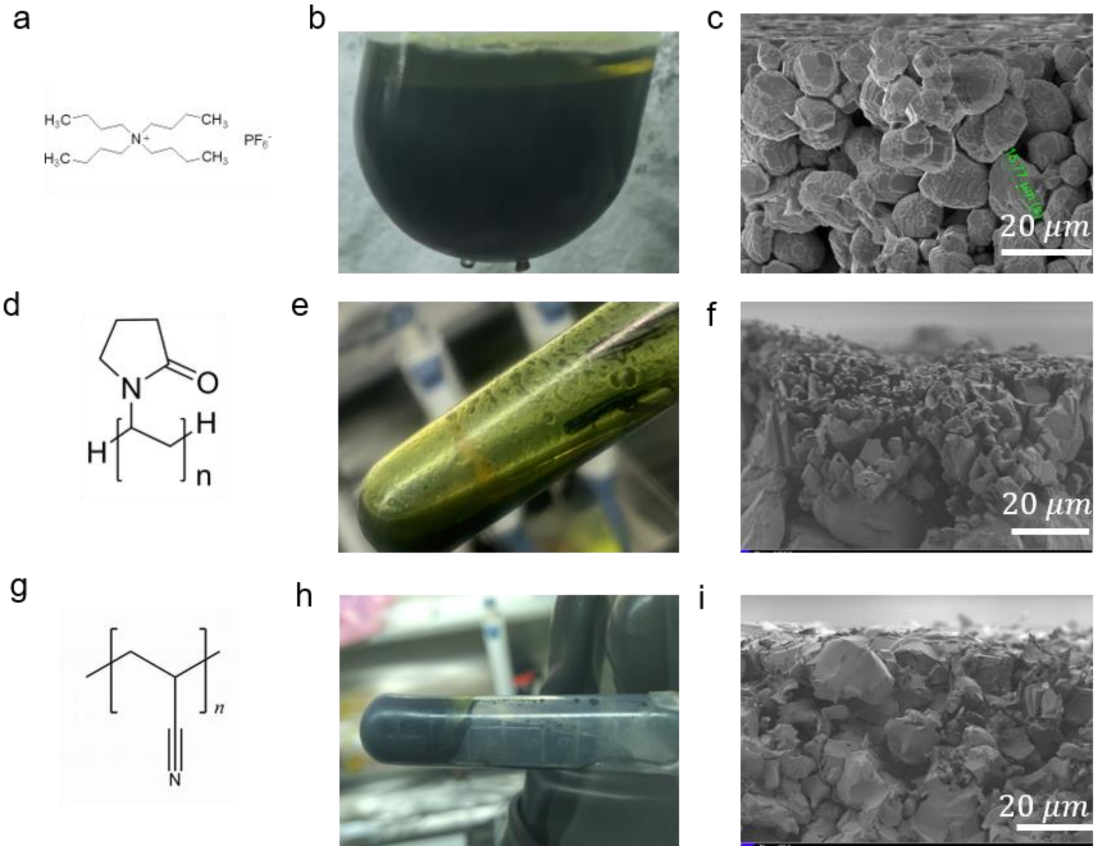

Figure S3. The ligands and their stabilization performance. The chemical formula of three types of ligands TAHF (a), PVP (d) and PAN (g). The suspension states and fabricated films after adding each ligands (b, c) for TAHF, (e, f) for PVP, (h, i) for PAN.

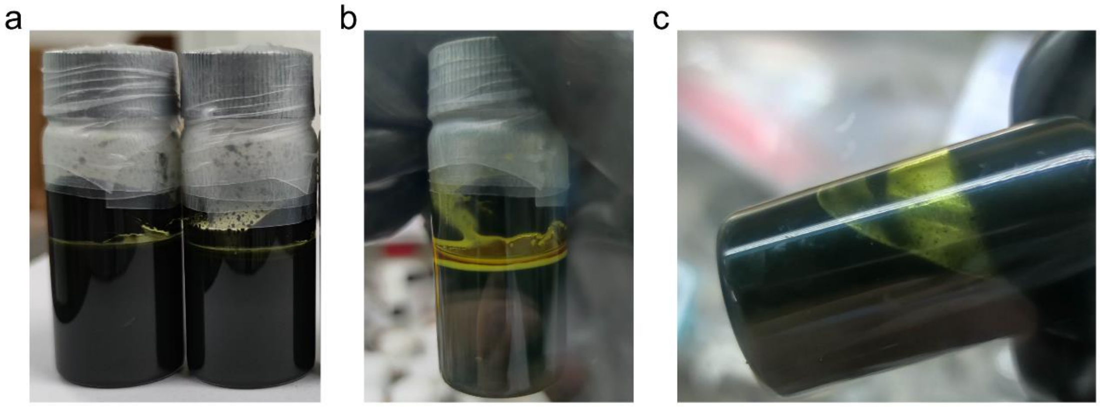

Figure S4. The different types of perovskite suspension (a) Fresh pristine suspension (left) and fresh storage-grade suspension prepared by ball milling (right). (b) Pristine suspension stored for 3 weeks. (c) storage-grade suspension stored for 3 weeks.

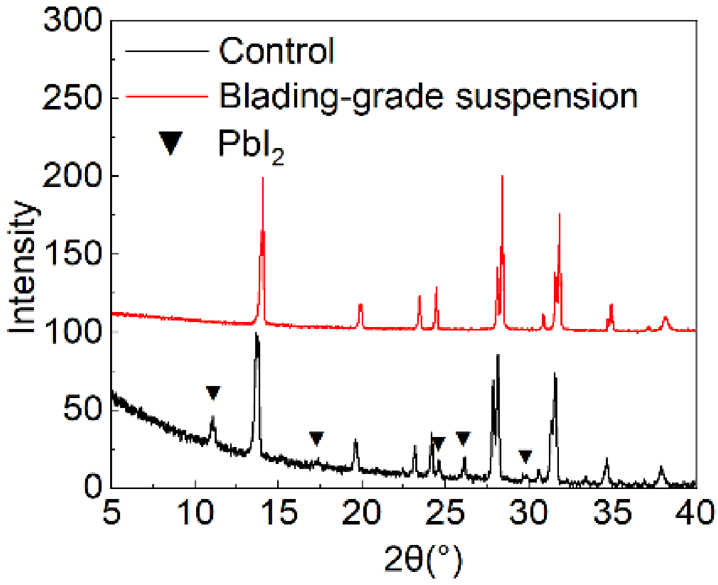

Figure S5. X-ray diffraction patterns of films prepared with pristine and blading-grade suspensions.

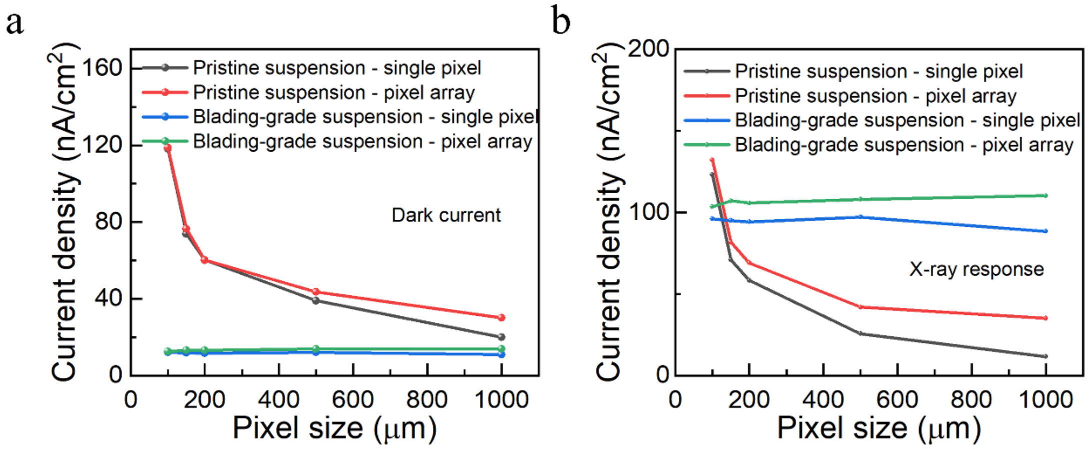

Figure S6. The pixel level uniformity data of the perovskite films. (a) The measured dark current densities for different pixel sizes. (b) The measured X-ray response current densities for different pixel sizes.

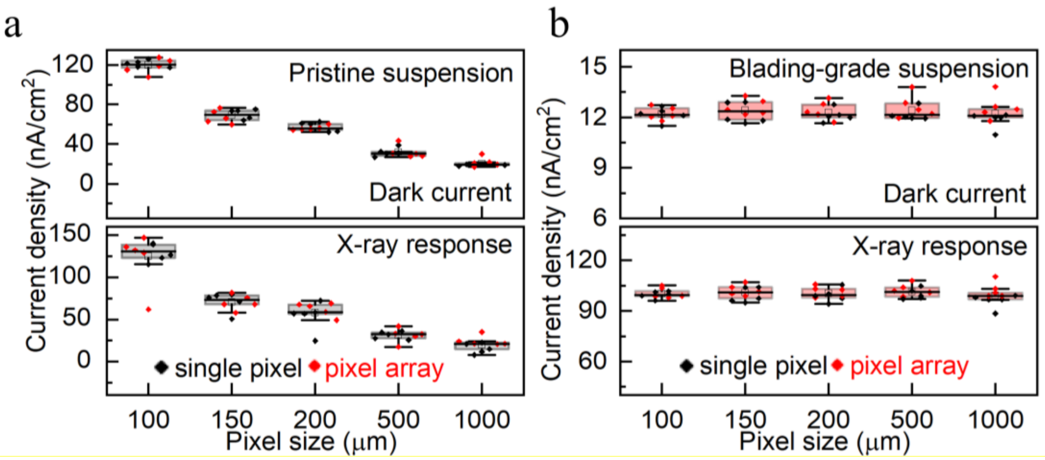

Figure S7. The statistical pixel level uniformity data of the perovskite films. (a) The statistical dark current densities and X-ray response current densities distribution for films from pristine suspension. (b) The statistical dark current densities and X-ray response current densities for films from blading-grade suspension.

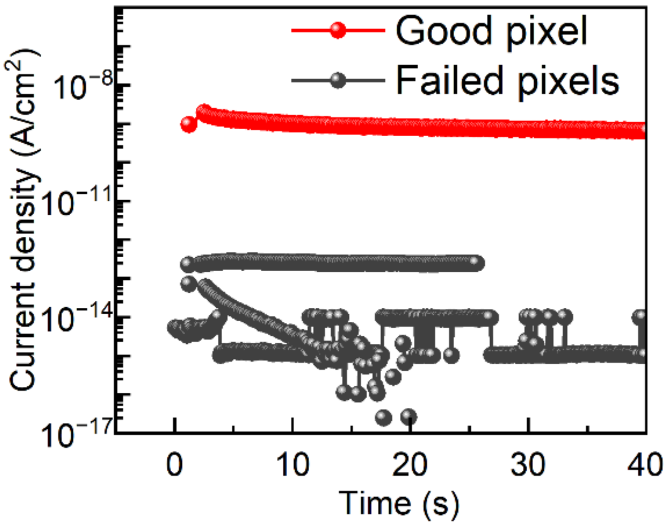

Figure S8. Time dependent response of 100 μm pixels with good contact and failed contacts.

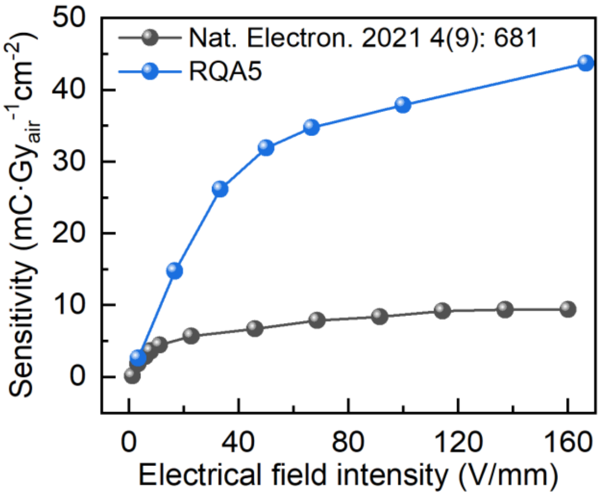

Figure S9. The comparison of sensitivity of the device from blading-grade suspension with the reference results (Nature Electronics, 2021, 4, 681) under the standard RQA5 spectrum.

Supplementary Note 1. Calculation process of the diffusion-based resolution limit.

Modulation transfer function (MTF) is the Fourier transform of the point spread function (p(x), that is, the image of a point source).[1] The theoretical pixel response is shown in Figure S10a.

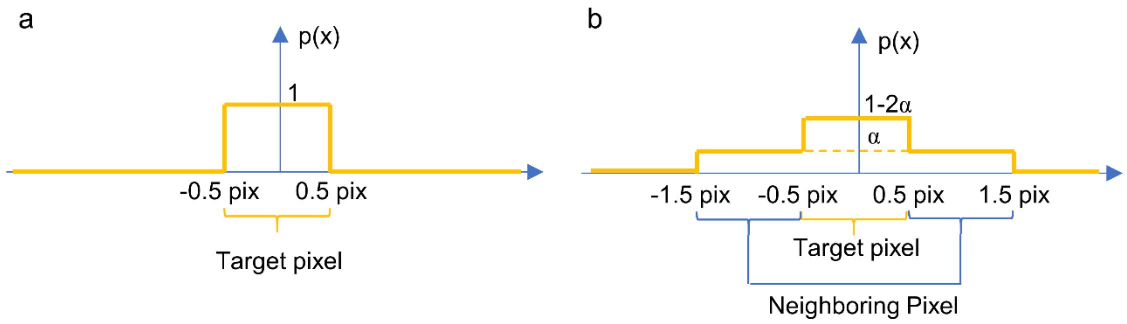

Figure S10. The 1D point spread function with/without diffusion. (a) The point spread function of pixels at an ideal condition. (b) The point spread function of pixels when the charge diffusion between neighboring pixel happens.

The theoretical pixel response p(x) could be expressed as:

$$\mathrm{p(x)} = \begin{cases} 1, |x| < 0.5pix \\ 0, |x| \geq 0.5\mathrm{pix} \end{cases} \qquad \text{(equation 1)}$$

The modulation transfer function (MTF) of pixels are as followings:

$$\mathrm{MTF}(\nu) = |\mathcal{F}\{\mathrm{p(x)}\}| = \int_{-\infty}^{+\infty} \mathrm{p(x)} \mathrm{e}^{-\mathrm{i}2\pi\nu\mathrm{x}} dx, \text{(equation 2)}$$

where ν is the spatial frequency (lp/pixel) and $\mathscr{F}$ represents the Fourier transform.

Then the maximum MTF of the pixel arrays is well known and is given by the sinc function:

$$\mathrm{MTF}(\nu) = \mathrm{sinc}(\nu) = \frac{\sin(\nu\pi)}{\nu\pi} \qquad \text{(equation 3)}$$

However, it is necessary to consider the charge sharing effects between the neighboring pixels on the theoretical resolution limit. The charge sharing in X-ray detectors includes the generation of the electron-hole pairs, reabsorption of K fluorescence and diffusion of the charge carriers during their drift towards the electrode[2]. As calculated in our previous study[3], the charge diffusion dominates the charge sharing effect compared to the primary

electron-hole pairs distribution and fluorescence effect. The charge diffusion is caused by the gradient in the carrier density, while the carriers move towards the surface under the influence of the applied electric field in the vertical direction. The carrier distribution caused by diffusion can be described according to the following relation[3]:

$$n(x) = n_0 \exp(-\frac{(x-x_0)^2}{s^2}) \quad \text{(equation 4)}$$

where $s^2 = 4Dt$, x_0 is the incident point of X-ray. In this equation, D is the diffusion constant ($D = kT\mu_i/e$, where k is the Boltzmann constant, T is the temperature, μ is the carrier mobility, and e is the electron charge). The transit time is given by the relation: $t = d/\mu_i E$, where d is the thickness of the detector, and E is the electric field applied to the detector. The charge diffusion leads to the response decrease of the given pixel and increase of the neighboring pixels, as shown in Figure S10b. The parameter α is the increased response of the neighboring pixels, which is proportional to the above calculated carrier distribution n(x). Then the theoretical p(x) could be amended as:

$$p(x) = \begin{cases} 1-2\alpha, |x| \le 0.5pix \\ \alpha, 0.5pix < |x| < 1.5pix \\ 0, |x| \ge 1.5pix \end{cases} \quad \text{(equation 5)}$$

The theoretical modulation transfer function (MTF) considering charge diffusion effect could be amended as:

$$\mathrm{MTF}(\nu) = |\mathcal{F}\{\mathrm{p(x)}\}| = \int_{-1.5pix}^{-0.5pix} \alpha e^{-i2\pi\nu x} dx + \int_{0.5pix}^{1.5pix} \alpha e^{-i2\pi\nu x} dx + \int_{-0.5pix}^{0.5pix} (1-2\alpha) e^{-i2\pi\nu x} dx$$

$$= \left[\frac{\alpha e^{i\pi\nu}}{-i2\pi\nu} - \frac{\alpha e^{i3\pi\nu}}{-i2\pi\nu}\right] + \left[\frac{\alpha e^{-i3\pi\nu}}{-i2\pi\nu} - \frac{\alpha e^{-i\pi\nu}}{-i2\pi\nu}\right] + \left[\frac{(1-2\alpha) e^{-i\pi\nu}}{-i2\pi\nu} - \frac{(1-2\alpha) e^{i\pi\nu}}{-i2\pi\nu}\right]$$

$$= (1-3\alpha)\frac{\sin(\pi\nu)}{\pi\nu} + \alpha\frac{\sin(3\pi\nu)}{\pi\nu} \quad \text{(equation 6)}$$

Equation 6 is the final state for the theoretical spatial resolution by considering the charge diffusion effect. In specific cases, the parameter α has to be calculated according to the pixel size, electric field and film thickness, as shown in equation 4.

The parameter α could be given as:

$$\alpha = \frac{p_N}{p_T + 2p_N} \quad \text{(equation 7)}$$

where p_T and p_N is the response value of the target and neighboring pixels, respectively. According to equation 4, p_T and p_N could be expressed as followings:

$$p_N \propto \int_{-1.5pix}^{-0.5pix}\int_{-0.5pix}^{0.5pix} n_0 \exp(-\frac{(x-x_0)^2}{s^2})dx_0dx \qquad \text{(equation 8)}$$

$$p_T \propto \int_{-0.5pix}^{0.5pix}\int_{-0.5pix}^{0.5pix} n_0 \exp(-\frac{(x-x_0)^2}{s^2})dx_0dx \qquad \text{(equation 9)}$$

Then we could derive the parameter α for the previous perovskite X-ray FPDs and this work according to the pixel size, thickness and electric field (Figure S11a).

For spatial resolution, we adopted the typical MTF value of 0.2 to determine the spatial frequency value according to equation 6. Hence, the diffusion-based resolution limit of the previous studies and our work is 0.568 lp/pix (Nature, 2017, 550, 87), 0.482 lp/pix (Nat Electron, 2021, 4, 681) and 0.56 lp/pix (this work).

a

Ref	Pixel size (μm)	Thickness (μm)	Electrical field intensity (V/mm)	alpha	Theoretical resolution (lp/pix)
Nature,2017,550,87	70	830	60	0.1667	0.568
Nat. Electron.,2021,4,681	50	230	30	0.1712	0.482
Our work	150	588	8.5	0.1670	0.560

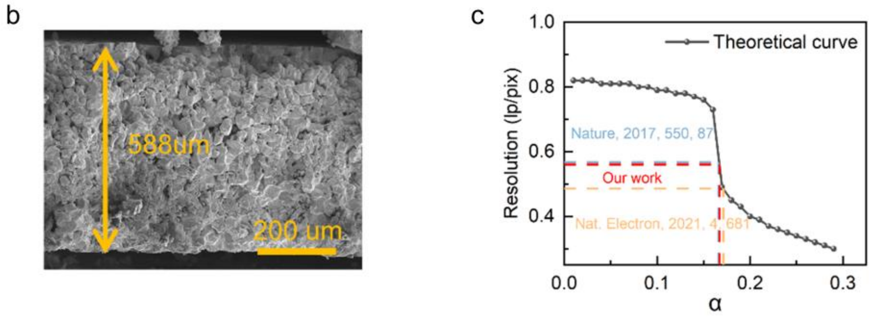

Figure S11. The diffusion-based resolution limit data of previous studies and our work. (a) The summary of key parameters for perovskite X-ray FPDs. (b)Cross-sectional SEM image of X-ray detector. (c) The charge-diffusion determined resolution limit at MTF=0.2.

Supplementary Note 2. μτ product derived from Hecht equation fitting.

Photoconductivity measurement was carried out on the uniform perovskite and nonuniform perovskite thick film with the structure of Au/perovskite/ITO. The μτ product was obtained by fitting with a modified Hecht equation[2,3]:

$$I = \frac{I_0 \mu\tau V}{L^2} \frac{1 - exp\left(-\frac{L^2}{\mu\tau V}\right)}{1 + \frac{L}{V}\frac{s}{\mu}}$$

where I_0 is the saturated photocurrent, L is the perovskite thick film thickness, V is the applied bias, τ is the carrier lifetime, μ is carrier mobility.

Supplementary Note 3. The calculation of Electron-hole pair creation energy and photoconductive gain.

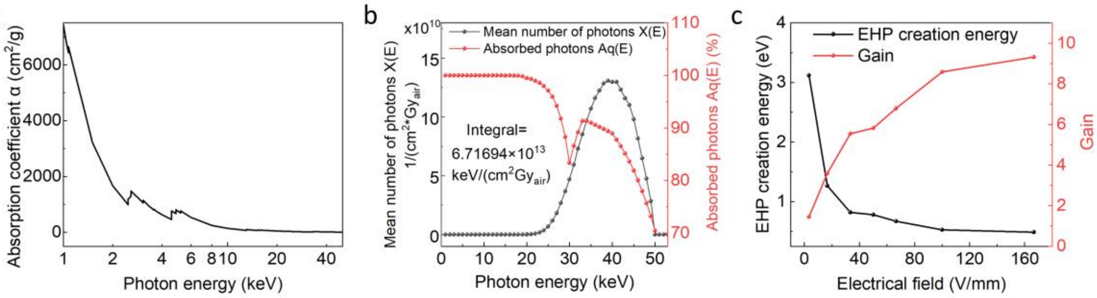

Figure S12. The attenuation & absorption curves of X-ray photons and the EHP creation energy & Gain of the detectors in this work. (a) The attenuation coefficient of $MAPbI_3$. (b) The simulated X-ray spectrum with RQA3 filtration (black line) and the percentage of absorbed photons vs photon energy (red). (c) The EHP creation energy and the gain of film from blading-grade suspension.

The electron–hole pair(EHP) creation energy W can be calculated according to the empirical model by Devanathan and his co-authors[4,5]

$$W=2E_g+1.43$$

The electron–hole pair creation energy of $MAPbI_3$ should be 4.53 eV.

To derive the gain factor, we have to obtain the absorbed energy for the detector.

$$Absorbed\ energy = \int X(E) * Aq(E)dE$$
$$Aq = 1 - \exp(\alpha\rho L)$$

In which, the function Aq(E) was the percentage of absorbed photons (Figure S12b), which was calculated from the total attenuation coefficient α for $MAPbI_3$ taken from the NIST XCOM cross-section database (Figure S12a). The function X(E) was the simulated X-ray spectrum with a RQA3 filtration (Figure S12b).

The gain can be calculated with the method documented in our previous work[6],

$$G = \frac{I_R}{I_p} = \frac{J_R/(Dose\ rate)}{J_p/(Dose\ rate)} = \frac{S_R}{S_p}$$

$$S_p = \frac{Absorbed\ energy}{W_\pm} \times e$$

$$= \frac{6.71694 \times 10^{13} keV \cdot cm^{-2} \cdot Gy_{air}^{-1}}{4.53 eV} \times 1.602 \times 10^{-19} C$$

$$= 2375.39 \mu C \cdot Gy_{air}^{-1} \cdot cm^{-2}$$

In which, I_R, I_p represent the real/theoretical photocurrent, J_R, J_p represent the real/theoretical photocurrent density, S_R, S_p represent the real/theoretical sensitivity. The theoretical sensitivity is 2375.39 $\mu C \cdot Gy_{air}{}^{-1} \cdot cm^{-2}$.

Supplementary Note 4. Calculation process for DQE curve.

According to the standard IEC 62220-1, the DQE can be calculated by,

$$DQE = \frac{\phi G^2 MTF^2(\mu,v)}{NPS(\mu,v)} = \frac{d^2 MTF^2(\mu,v)}{\phi\, NPS(\mu,v)} = \frac{MTF^2(\mu,v)}{K\chi(NPS(\mu,v))/d^2} = \frac{MTF^2(\mu,v)}{K\chi NNPS(\mu,v)}$$

where Φ (photons/mm^2) is the incident photon density, G is the gain of detector, MTF is the modulation transfer function, NPS is the noise power spectrum, d (Digitals) is the average gray scale of pixels, K (μGy) is the X-ray air kerma (or X-ray dose) and χ is the X-ray quanta per area per air kerma value of X-ray spectrum.

We conducted flat-field correction and averaged 100 images for calculation of NPS according to the standard IEC 62220-1. The NPS was derived from the 2D Fourier transformation of the averaged image. The normalized NPS was acquired by dividing the NPS with the power of the average gray scale as shown in **Figure S13a**. The X-ray quanta per area per air kerma value of X-ray spectrum could be given by the standard RQA spectrum (Table S1). Here we used RQA3 spectrum for imaging.

Then we could derive the DQE curve with air kerma 525.9 μGy, and the result was shown in **Figure S13b.** The comparison of DQE value of our detector with commercial product is summarized in Table S2.

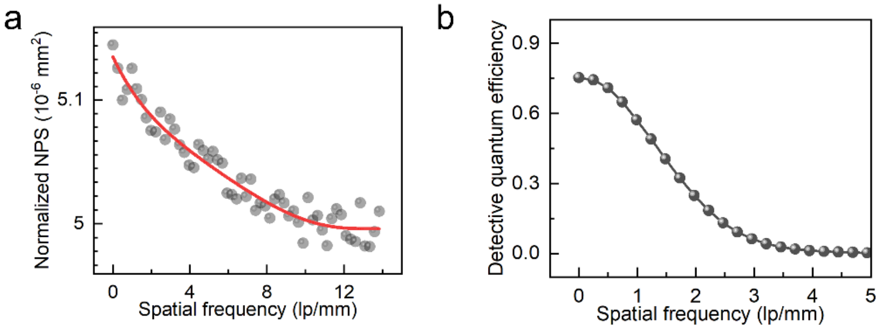

Figure S13. The NPS and DQE curves of the detectors in this work. (a)The normalized noise power spectrum of the image from the device prepare with blading-grade suspension. (b) The detective quantum efficiency of the device prepared with blading-grade suspension.

Table S1. X-ray Quanta per area per air kerma for different spectrum.

Spectrum	Quanta per area per air kerma (photons/(mm2 ·µGy))
RQA3	21759
RQA5	30174
RQA7	32362
RQA9	31077

Table S2. DQE comparison of our work and commercial detectors.

Detector	Pixel pitch	DQE at 0lp/mm	DQE at 1 lp/mm
Our work	150 µm	75.3%	57%
GC85A, Samsung	140µm	80 %	None
PaxScan 4343DXV, Varex	139 µm	78 %	None
4343RF, Varex	150µm	None	62%

Supplementary References

1. Fliegel, K. Modeling and measurement of image sensor characteristics. *Radioengineering* 13, 27–34 (2004).

2. Many, A. High-field effects in photoconducting cadmium sulphide. *J. Phys. Chem. Solids* 26, 575–578 (1965).

3. Zhou, Y. et al. Heterojunction structures for reduced noise in large-area and sensitive perovskite x-ray detectors. *Sci Adv* 7, eabg6716 (2021).

4. Devanathan, R., Corrales, L. R., Gao, F. & Weber, W. J. Signal variance in gamma-ray detectors—A review. *NUCL INSTRUM METH A* **565**, 637–649 (2006).

5. Wei, H. et al. Sensitive X-ray detectors made of methylammonium lead tribromide perovskite single crystals. *Nat. Photon.* 10, 333–339 (2016).

6. Pan, W. et al. $Cs_2AgBiBr_6$ single-crystal X-ray detectors with a low detection limit. *Nat. Photon.* 11, 726–732 (2017).
